# Association between short-term exposure to atmospheric black carbon and acute exacerbations of childhood asthma

**DOI:** 10.3389/fped.2026.1756335

**Published:** 2026-05-26

**Authors:** Yating He, Zhonghua Lu, Wanting Huang, Zhimeng Huang, Jinzhun Wu

**Affiliations:** 1 Department Pediatrics, Women and Children’s Hospital, School of Medicine, Xiamen University, Xiamen, Fujian, China; 2 School of Public Health, Xiamen University, Xiamen, Fujian, China

**Keywords:** acute exacerbation, air pollution, black carbon, childhood asthma, short-term exposure

## Abstract

**Objective:**

This study aimed to investigate the association between short-term exposure to atmospheric black carbon and acute exacerbations of childhood asthma and provide a theoretical basis for identifying strategies to reduce air pollutant exposure and prevent acute asthma attacks in children.

**Methods:**

Data on chilhood asthma cases were obtained from the Xiamen Health and Medical Big Data Center between January 2020 and December 2023. A case-crossover design was applied, with the day of acute asthma exacerbation designated as the case day and the 7 days prior designated as control days. The study collected data on black carbon, PM₂.₅, and its components (sulfate, nitrate, ammonium, and organic matter) concentrations, as well as meteorological factors for the 0–6 day lag period. Generalized linear mixed-effects model (GLMM) were used to develop single- and two-pollutant models, while Bayesian kernel machine regression (BKMR) was employed to build multi-pollutant models. These models were used to evaluate the independent, interactive, and combined effects of short-term black carbon exposure. Stratified analyses were conducted by age, sex, and season.

**Results:**

A total of 3,440 cases were ultimately included in this study. After adjusting for temperature, relative humidity, fever, and total PM₂.₅ mass, the single-pollutant model revealed that black carbon exposure was significantly associated with acute asthma exacerbations at a 3-day lag (aOR = 1.2089, 95% CI: 1.0348–1.4122), with the risk increasing as black carbon concentrations rose. In the two-pollutant model, black carbon exposure at a 3-day lag remained significantly associated with acute asthma exacerbations and demonstrated interaction effects with PM_2.5_ components including sulfate, nitrate, and ammonium. In the multi-pollutant model, mixed exposure to pollutants was positively associated with acute asthma exacerbations at a 3-day lag, with black carbon emerging as a critical factor. Additionally, black carbon exhibited interaction effects with ammonium. Stratified analysis indicated that black carbon exposure in winter was more likely to trigger acute asthma exacerbations.

**Conclusion:**

Short-term exposure to black carbon is significantly associated with acute asthma exacerbations in children, particularly at a 3-day lag. The risk is higher in children exposed in winter. Furthermore, complex interaction effects exist between black carbon and other pollutants.

## Introduction

Asthma is one of the most common chronic respiratory diseases in childhood. Epidemiological surveys conducted in urban areas of China indicate a rising trend in cumulative prevalence, with rates of 1.00% (1), 1.97% (2), and 3.02% (3), reported in three consecutive studies. Based on clinical presentation, asthma can be classified into acute exacerbation, chronic persistent, and clinical remission phases (4). The primary clinical goal in asthma management is to achieve and maintain effective disease control. However, the risk factors influencing childhood asthma onset, progression, and exacerbations are complex. These factors include a personal history of allergies (5–8), family history of asthma (7, 8), atopic dermatitis and/or allergic rhinitis (9), history of wheezing (9), elevated IgE levels (9), exposure to air pollutants such as PM₂.₅, black carbon, nitrogen dioxide, and sulfur dioxide (10–12), tobacco smoke (9), acute upper respiratory infections (5, 7, 8), and obesity (13). Despite advances in asthma management, overall disease control remains suboptimal. Among these risk factors, air pollutants, particularly PM₂.₅, exert multiple adverse effects on children's respiratory health. A study estimated that 13% of childhood asthma cases globally could be attributed to traffic-related air pollution (14), highlighting the need for further research on the impact of air pollutants on childhood asthma.

Black carbon (BC) is a major component of PM₂.₅, with primary particle sizes ranging from 10 to 100 nm. It is produced through the incomplete combustion or pyrolysis of carbonaceous materials (e.g., coal, natural gas, and fuel oil) under conditions of limited oxygen availability (15–17). The World Health Organization (WHO) Global Air Quality Guidelines (AQG) highlight that both short-term and long-term exposure to BC is associated with cardiovascular health effects and premature mortality, making it a significant risk factor for human health (18). Studies have shown that long-term exposure to PM₂.₅ and its components—including BC, sulfate (SO₄²⁻), nitrate (NO₃⁻), ammonium (NH₄⁺), and organic matter (OM)—is significantly associated with asthma prevalence in adults, with BC exhibiting the strongest association (19). Additionally, both prenatal and childhood exposure to BC have been positively correlated with the incidence of childhood asthma (12). A population-based prospective cohort study demonstrated that a decline in PM₂.₅ exposure from school age to young adulthood was associated with a reduced incidence of asthma. Although no significant association was observed for BC, a decreasing trend in asthma incidence was noted with lower BC levels (20).

Previous studies have established a link between long-term BC exposure and asthma onset. However, research on the association between short-term BC exposure and acute exacerbations of childhood asthma remains limited. This study aims to assess this association by analyzing childhood asthma exacerbation cases using a case-crossover design. To account for potential lag effects and interactions with other pollutants, we employ generalized linear mixed-effects model (GLMM) to construct single- and two-pollutant models and Bayesian kernel machine regression (BKMR) for multi-pollutant modeling. Furthermore, stratified analyses based on age, sex, and season are conducted. The findings of this study will contribute to identifying strategies for reducing air pollutant exposure and informing clinical interventions to prevent acute asthma exacerbations in children.

## Methods

### Study population

This study collected outpatient and emergency medical records of childhood asthma cases from the Xiamen Health and Medical Big Data Center, which integrated data from 19 general hospitals and 39 community health centers, between January 2020 and December 2023. The records included patient ID, sex, date of birth, residential address, visit date, chief complaint, present illness history, past medical history, personal history, family history, physical examination findings, and confirmed diagnosis. Asthma cases were identified using the International Classification of Diseases (ICD-10) code J45. Based on the *Guideline for the diagnosis and optimal management of asthma in children (2016)*, cases with a sudden onset (disease duration ≤7 days) of wheezing, cough, shortness of breath, chest tightness, or a rapid exacerbation of pre-existing symptoms were defined as acute asthma exacerbations, while cases were excluded if they involved medication self-discontinuation within 14 days, exercise, allergen exposure, or unknown dates of acute exacerbation, in order to reduce potential confounding and better isolate the effects of ambient air pollution. The specific inclusion and exclusion criteria were as follows:

Inclusion criteria: (1) Children diagnosed with acute asthma exacerbation between 2020 and 2023; (2) Complete medical records, including residential address information; (3) Residence in Xiamen.

Exclusion criteria: (1) Cases involving medication self-discontinuation within 14 days, exercise, allergen exposure; (2) Cases with unknown dates of acute exacerbation.

To minimize bias from repeated visits, we defined independent asthma exacerbation events using a washout period of 7 days between consecutive visits for the same patient. Only the first visit within each episode cluster was included in the analysis.

### BC, PM₂.₅, and component data

Daily average concentrations of BC, PM₂.₅, and its components (SO₄²⁻, NO₃⁻, NH₄⁺, and OM) were obtained from the Tracking Air Pollution in China (TAP) dataset (http://tapdata.org.cn/), developed by Tsinghua University in collaboration with Peking University, Nanjing University, Fudan University, and the Chinese Academy of Meteorological Sciences. This dataset provides nationwide air pollution estimates at a spatial resolution of 10 × 10 km, allowing precise matching of pollutant exposure levels to the geographic coordinates of each patient's residence.

The modeling details of the TAP dataset have been described in previous studies (21–26). Briefly, TAP integrates multiple data sources, including PM₂.₅ monitoring data, satellite-derived aerosol optical depth (AOD), community multiscale air quality (CMAQ), meteorological reanalysis, land use data, altitude data, and population data, using a two-stage machine learning framework. In the first stage, a high-pollution event index was defined based on observational data, and the synthetic minority oversampling (SMOTE) technique was applied to the training dataset to improve representation of high-pollution events. A random forest model was then trained to predict pollution levels. In the second stage, a second random forest model was trained using residuals between observed and CMAQ-simulated PM₂.₅ concentrations, which enhanced model responsiveness to PM₂.₅ variations. Decision-tree-based methods were used to impute missing satellite data. Chemical component concentrations were estimated by constraining total PM₂.₅ mass and applying corrections based on CMAQ outputs. To refine CMAQ model outputs, the dust emission module was improved, and an extreme gradient boosting (XGBoost) algorithm was used to adjust the relative contributions of PM₂.₅ components based on observational data, ensuring more accurate estimates of PM₂.₅ component concentrations.

### Meteorological data

Meteorological data, including daily average temperature (°C) and relative humidity (%), were obtained from the National Meteorological Science Data Center (https://data.cma.cn/) for the period from 2020 to 2023 in Xiamen.

### Study design

A unidirectional case-crossover design was employed, with asthma exacerbations serving as the outcome variable. The day of acute exacerbation was designated as the case day, while the seven preceding days were used as control days to account for intra-individual correlations and potential weekday effects. Considering potential lag effects of BC exposure, daily average concentrations of BC and other air pollutants were traced back up to six days before each exacerbation event to assess their influence.

### Statistical analysis

Spearman correlation analysis was used to evaluate relationships between air pollutants and meteorological factors. After adjusting for daily average temperature, relative humidity, fever, and total PM₂.₅ mass, generalized linear mixed-effects model (GLMM) with a binomial distribution with a logit link function was employed to develop single- and two-pollutant models, in which the occurrence of acute asthma exacerbation (yes/no) was treated as the dependent variable and BC concentration was included as the independent variable. Patient identification number was modeled as a random effect to account for within-subject correlation arising from repeated visits. Distributed lag non-linear model (DLNM) was applied to assess lagged exposure effects over lag days 0–6. Variance inflation factors (VIF) were calculated to assess multicollinearity among pollutants. Although a threshold of VIF < 10 was used to indicate the absence of severe multicollinearity, moderate collinearity among PM₂.₅ components may still affect the precision and interpretability of models. Therefore, to account for the potential multicollinearity and complex correlations among air pollutants, we further applied Bayesian kernel machine regression (BKMR) to develop multi-pollutant model, which allows flexible estimation of non-linear and interactive relationships within correlated pollutant mixtures. Statistical analyses were performed using R software (version 4.4.1), with a significance threshold of *P* < 0.05.

## Results

### General characteristics of the study population

**Figure 1:**
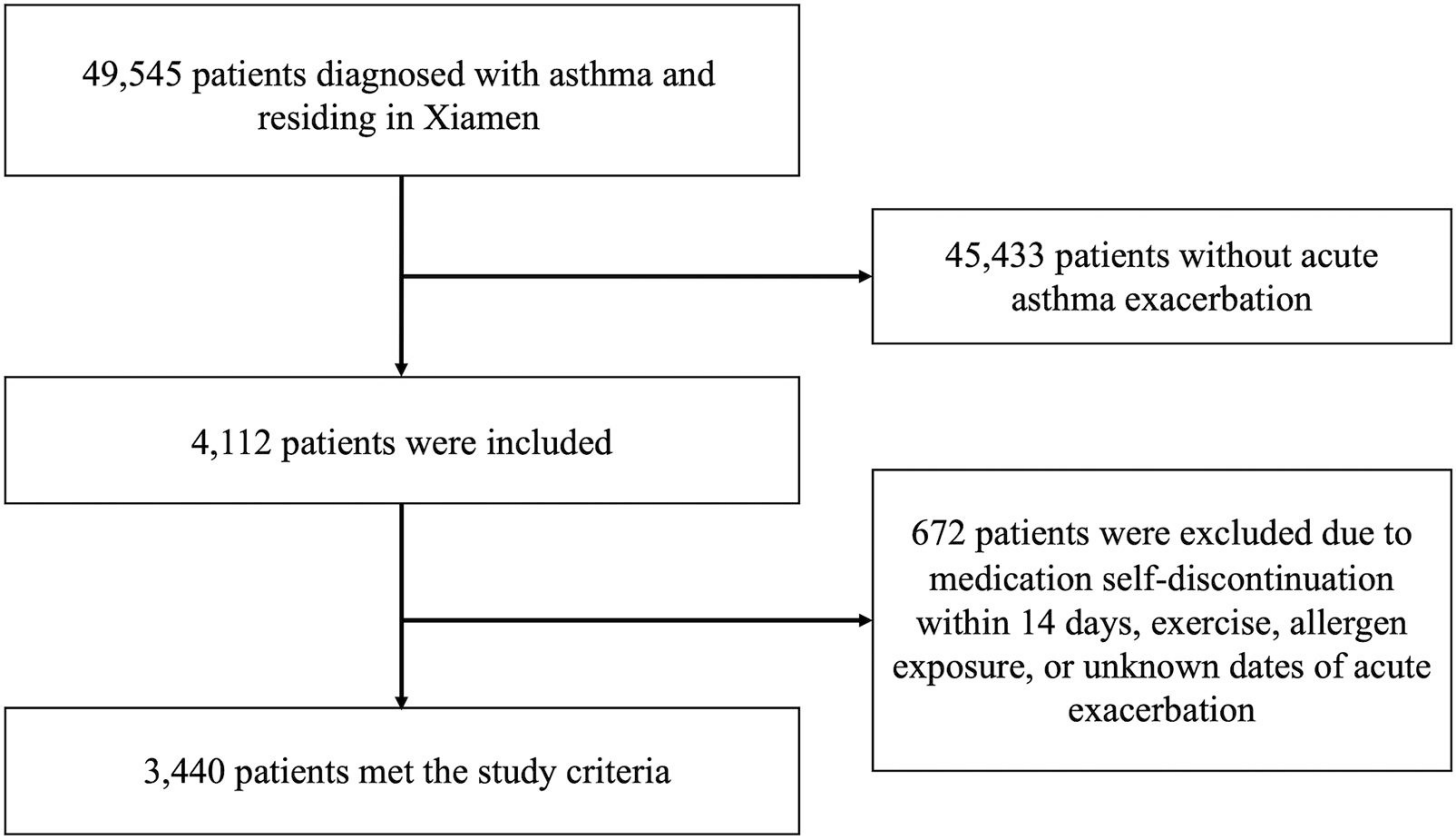
Flowchart of inclusion and exclusion criteria.

From January 2020 to December 2023, a total of 49,545 outpatient and emergency cases were recorded at the Xiamen Health and Medical Big Data Center with an asthma diagnosis coded as J45 and a registered residential address in Xiamen. Based on the *Guideline for the diagnosis and optimal management of asthma in children (2016)*, 4,112 cases of acute asthma exacerbation were included. After excluding 672 cases due to medication self-discontinuation within 14 days, exercise, allergen exposure, or unknown dates of acute exacerbation, a final total of 3,440 cases of acute asthma exacerbation were included for subsequent analysis (Figure 1).

Among the 3,440 cases, 2,373 (68.98%) were male and 1,067 (31.02%) were female. Children under six years old accounted for 56.22% of the cases. Additionally, 40.06% of the patients had comorbid allergic rhinitis and/or atopic dermatitis, 21.60% had a history of eczema, and 874 cases (25.41%) had a family history of allergic diseases (Table 1).

**Table 1:** Characteristics of acute asthma exacerbation cases from 2020 to 2023.

Characteristics	Mean ± SD or *n* (%)
Age (years old)	5.4 ± 2.4
<6 years old	1,934 (56.22)
≥6 years old	1,506 (43.78)
Sex	
Male	2,373 (68.98)
Female	1,067 (31.02)
Allergic rhinitis and/or atopic dermatitis	1,378 (40.06)
History of eczema	743 (21.60)
Family history of allergic diseases	874 (25.41)
Season	
Spring	887 (25.78)
Summer	736 (21.40)
Autumn	1,076 (31.28)
Winter	741 (21.54)

### Distribution of air pollutants and meteorological factors

Between 2020 and 2023, the mean daily exposure concentrations of PM₂.₅, BC, SO₄²⁻, NO₃⁻, NH₄⁺, and OM in Xiamen were 20.26 μg/m^3^, 1.29 μg/m^3^, 3.93 μg/m^3^, 2.95 μg/m^3^, 2.01 μg/m^3^, and 6.67 μg/m^3^. The mean daily average temperature and relative humidity were 21.95°C and 75.32% (Table 2).

**Table 2:** Distribution of daily air pollutants and meteorological factors in Xiamen from 2020 to 2023.

Air pollutants and meteorological factors	Mean	Min	Max	Q1	Q2	Q3	IQR^a^
PM_2.5_ (μg/m^3^)	20.26	1.00	84.00	12.60	18.00	26.00	13.40
BC (μg/m^3^)	1.29	0.03	7.02	0.79	1.15	1.64	0.85
SO₄²⁻ (μg/m^3^)	3.93	0.12	16.67	2.45	3.36	5.08	2.63
NO₃⁻ (μg/m^3^)	2.95	0.08	20.68	1.45	2.34	3.82	2.37
NH₄⁺ (μg/m^3^)	2.01	0.05	13.03	0.95	1.54	2.62	1.67
OM (μg/m^3^)	6.67	0.16	32.21	4.02	5.85	8.47	4.45
Temp^b^ (°C)	21.95	5.84	32.69	16.84	22.28	27.70	10.86
RH^c^ (%)	75.32	27.00	100.00	67.00	76.44	84.62	17.62

a IQR, interquartile range, IQR = Q3—Q1.

b Temp, daily average temperature (°C).

c RH, daily relative humidity (%).

### Correlation analysis of air pollutants and meteorological factors

**Figure 2:**
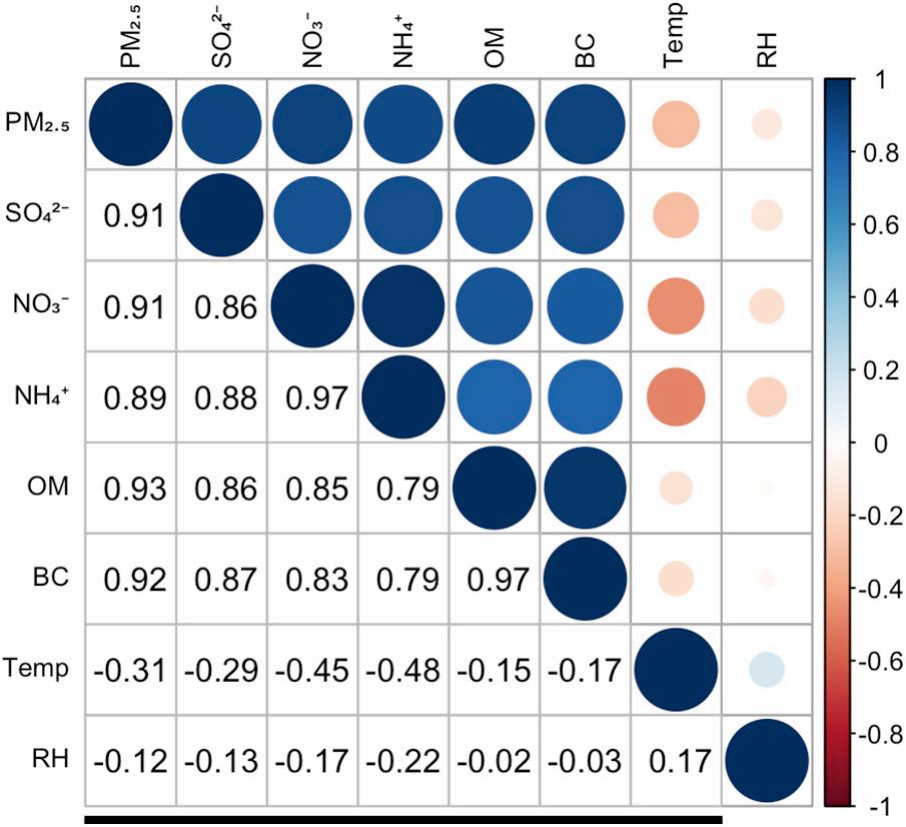
Correlation analysis of air pollutants and meteorological factors in Xiamen from 2020 to 2023.

Spearman correlation analysis was performed to evaluate the relationships between BC, PM₂.₅, its components, and meteorological factors. The results showed significant positive correlations between BC and PM₂.₅ as well as its components, with correlation coefficients exceeding 0.75 (*P* < 0.001). OM was positively correlated with relative humidity (*P* < 0.001), whereas all other air pollutants, except BC (*P* = 0.403), exhibited negative correlations with temperature and relative humidity (*P* < 0.001) (Figure 2).

### Single-pollutant model

**Figure 3:**
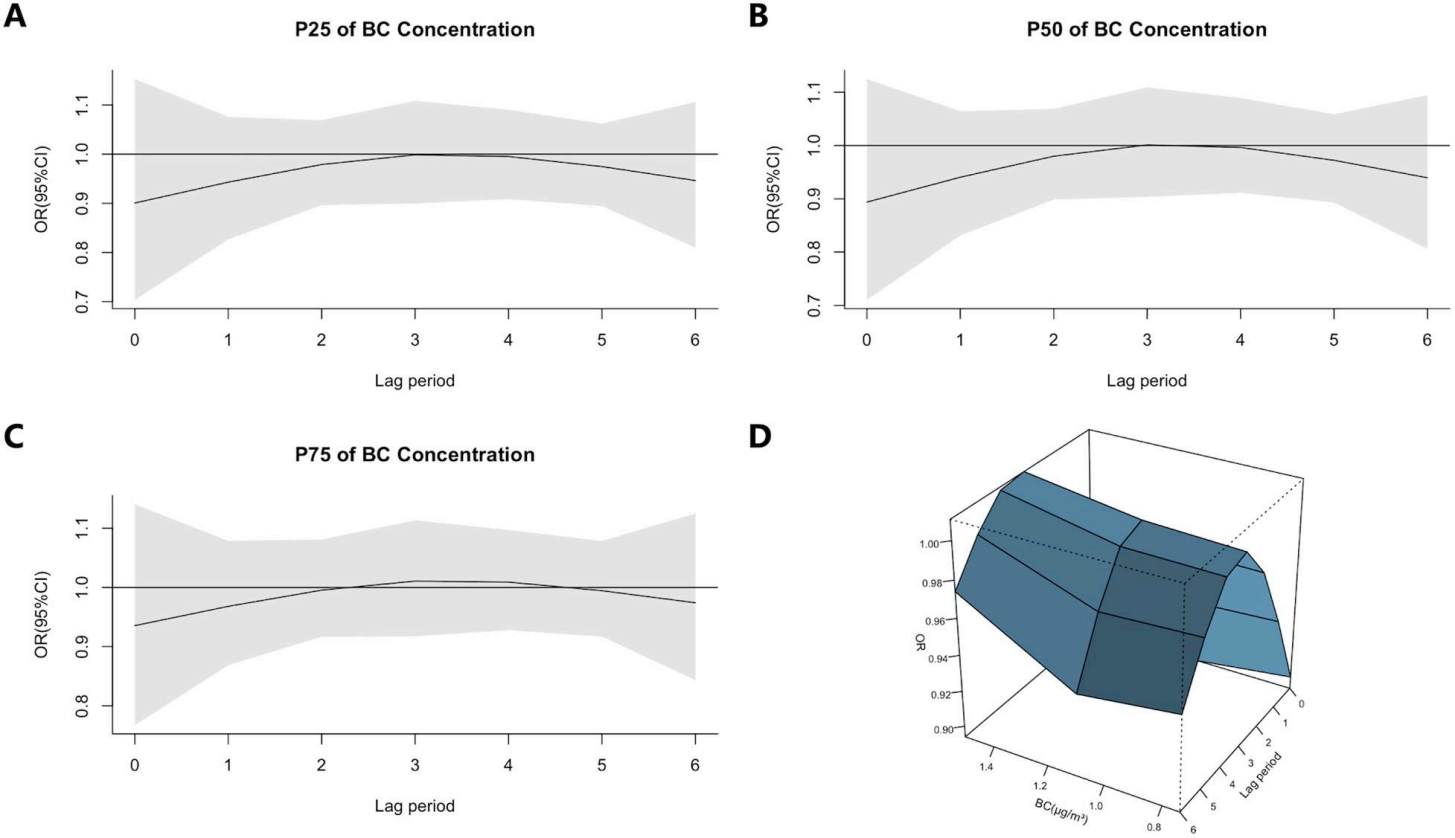
Lag effects of BC exposure at different concentrations on acute asthma exacerbation. **(A)** The lag effects of BC exposure at the 25th percentile (P25) on acute asthma exacerbation; **(B)** the lag effects at the 50th percentile (P50); **(C)** the lag effects at the 75th percentile (P75); **(D)** 3D plot of the lag effects of BC exposure on acute asthma exacerbation.

GLMM were used to develop single-pollutant models for each pollutant at lags of 0–6 days. The results indicated that BC exposure had the greatest effect 3 days before acute asthma exacerbation [adjusted odds ratio [aOR] = 1.2089, 95% confidence interval [CI]: 1.0348–1.4122] (Table 3). DLNM was further applied to explore the lag effect of short-term BC exposure, revealing an increasing effect up to 3 days post-exposure, followed by a decline. Additionally, higher BC concentrations were associated with an increased risk of acute asthma exacerbation (Figure 3).

**Table 3:** Association between BC and acute asthma exacerbation at different lag periods.

Lag period	aOR^a^	95% CI	*P*
0	0.9269	0.7966, 1.0784	0.409
1	1.0402	0.8941, 1.2101	0.668
2	1.0758	0.9216, 1.2557	0.437
3	1.2089	1.0348, 1.4122	0.045*
4	0.9649	0.8313, 1.1199	0.693
5	0.9362	0.8032, 1.0913	0.480
6	0.9916	0.8466, 1.1614	0.930

a aOR, odds ratio adjusted for daily average temperature, relative humidity, fever, and total PM₂.₅ mass.

* *P* < 0.05.

Among other pollutants, only NH₄⁺ exposure at lag0 showed a significant association with asthma exacerbations (aOR = 1.0887, 95% CI: 1.0268–1.1544) (Supplementary Table S1).

**Figure 4:**
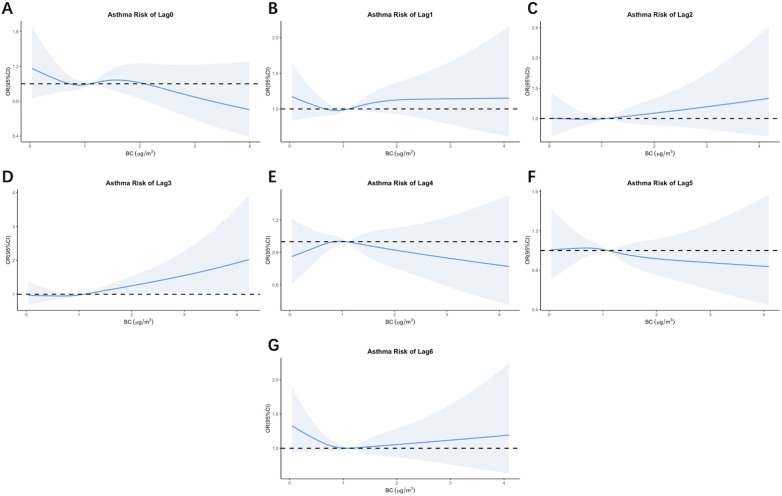
Exposure-response curves of BC and acute asthma exacerbation at different lag periods. **(A)** The exposure-response curve between BC and acute asthma exacerbation at a lag of 0 days; **(B)** at a lag of 1 day; **(C)** at a lag of 2 days; **(D)** at a lag of 3 days; **(E)** at a lag of 4 days; **(F)** at a lag of 5 days; **(G)** at a lag of 6 days.

A restricted cubic spline function (with four knots) was used to plot the exposure-response curve between BC and acute asthma exacerbations (Figure 4), demonstrating a linear positive correlation at a 3-day lag (*P* for Nonlinea*r* = 0.495).

### Two-pollutant model

GLMM were constructed for BC and other PM₂.₅ components at lags of 0–6 days, with VIF calculated. The results showed that at a 3-day lag, BC exposure remained significantly associated with acute asthma exacerbations in models including NO₃⁻ and NH₄⁺, with aOR values of 1.2421 (95% CI: 1.0460–1.4750) and 1.2355 (95% CI: 1.0416–1.4655) (Table 4). No significant associations were observed at other lag periods (Supplementary Table S2). VIF calculations indicated model stability for BC with SO₄²⁻, NO₃⁻, and NH₄⁺ (VIF < 10), whereas the BC-OM model was unstable (VIF > 10) and was excluded from further analysis.

**Table 4:** Association between two-pollutant exposure and acute asthma exacerbation at lags of 3 days.

Lag period	Two-pollutant (BC + X)	BC			X		
		aOR^a^	95% CI	*P*	aOR	95% CI	*P*
3	BC + SO₄²⁻	1.1517	0.9577, 1.3850	0.208	0.9765	0.9242, 1.0318	0.477
	BC + NO₃⁻	1.2421	1.0460, 1.4750	0.038*	1.0295	0.9640, 1.0994	0.467
	BC + NH₄⁺	1.2355	1.0416, 1.4655	0.042*	1.0320	0.9444, 1.1277	0.559
	BC + OM	1.2435	0.9658, 0.9907	0.156	0.9831	0.9266, 1.0431	0.341

a aOR, odds ratio adjusted for daily average temperature, relative humidity, fever, and total PM₂.₅ mass.

* *P* < 0.05.

**Figure 5:**
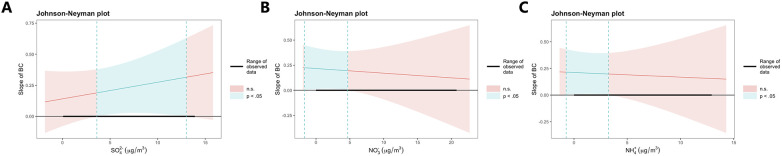
Interaction effects between BC and other PM₂.₅ components. **(A)** The interaction effect between SO₄²⁻ and BC at a lag of 3 days; **(B)** the interaction effect between NO₃⁻ and BC at a lag of 3 days; **(C)** the interaction effect between NH₄⁺ and BC at a lag of 3 days.

The Johnson-Neyman method was used to analyze potential interactions between BC and SO₄²⁻, NO₃⁻, and NH₄⁺. The results showed that at a 3-day lag, BC exposure was significantly associated with asthma exacerbations when SO₄²⁻ (3.60–13.00 μg/m³), NO₃⁻ (<4.68 μg/m³), and NH₄⁺ (<3.23 μg/m^3^) were within certain ranges. With increasing SO₄²⁻ levels, the risk of exacerbation increased (Figure 5).

At a 6-day lag, when SO₄²⁻ (>9.54 μg/m^3^), NO₃⁻ (>11.03 μg/m^3^), and NH₄⁺ (>6.90 μg/m^3^) exceeded threshold values, BC exposure was significantly associated with exacerbations, and higher concentrations of these pollutants further increased the risk. No significant interactions were observed at other lag periods (Supplementary Figure S1).

### Multi-pollutant model

**Figure 6:**
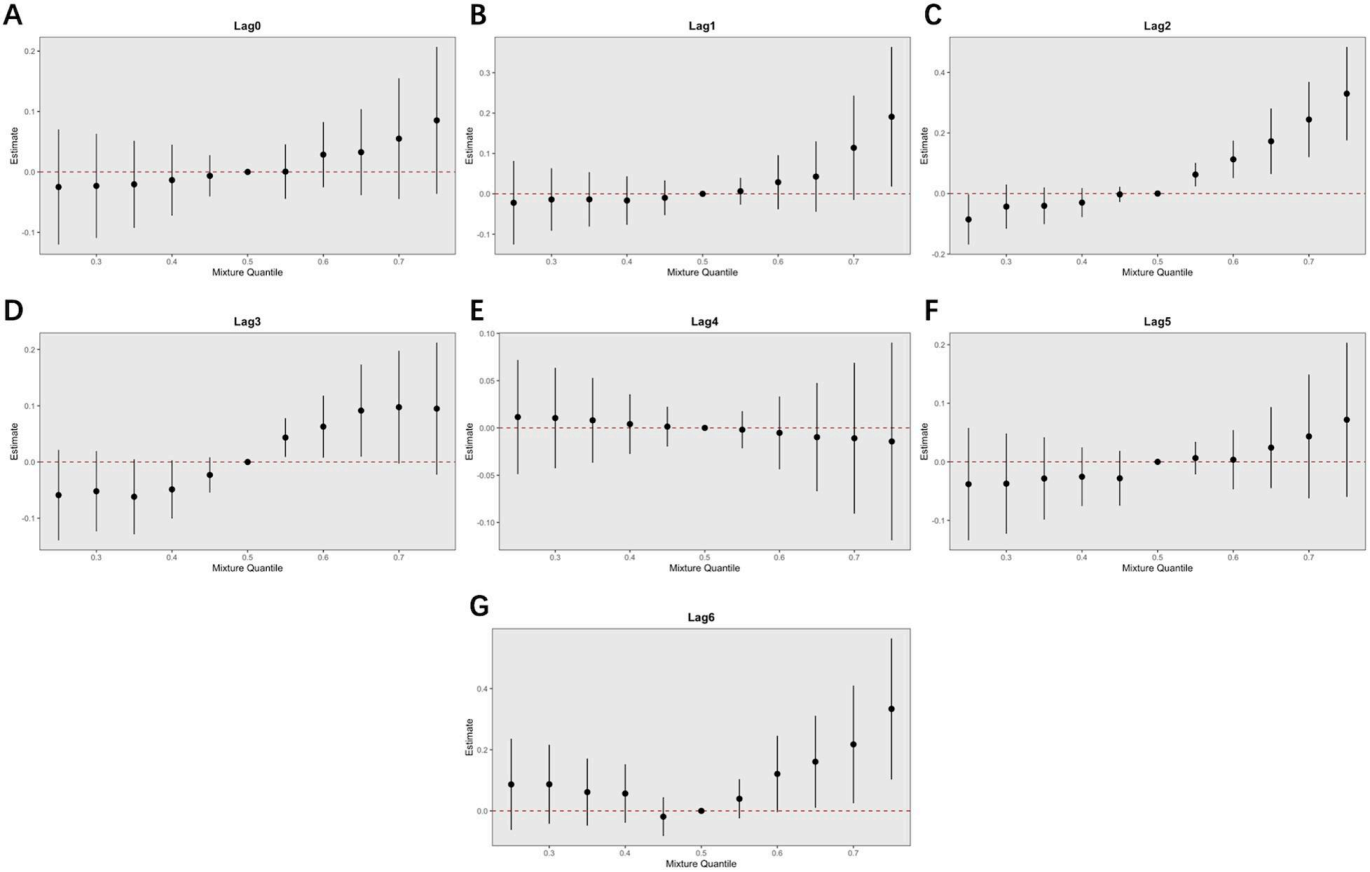
Mixed exposure effects of BC and other PM₂.₅ components at different lag periods. **(A)** The mixed exposure effects of BC and other PM₂.₅ components at a lag of 0 days; **(B)** at a lag of 1 day; **(C)** a lag of 2 days; **(D)** at a lag of 3 days; **(E)** at a lag of 4 days; **(F)** at a lag of 5 days; **(G)** at a lag of 6 days.

BKMR was used to construct multi-pollutant models at lags of 0–6 days. The results indicated a positive association between mixed exposure to BC and other PM₂.₅ components and acute asthma exacerbations at lags of 1, 2, and 3 days, with the strongest exposure effects observed at the 75th percentile (P75) (aOR = 1.2103, 95% CI: 1.0179–1.4391), P75 (aOR = 1.3908, 95% CI: 1.1916–1.6234), and the 65th percentile (P65) (aOR = 1.0956, 95% CI: 1.0094–1.1892). At a 6-day lag, exposure concentrations above the 50th percentile (P50) were positively associated with acute asthma exacerbations, with the peak exposure effect observed at P75 (aOR = 1.3960, 95% CI: 1.1082–1.7586). No significant associations were observed at other lag periods (Figure 6).

**Figure 7:**
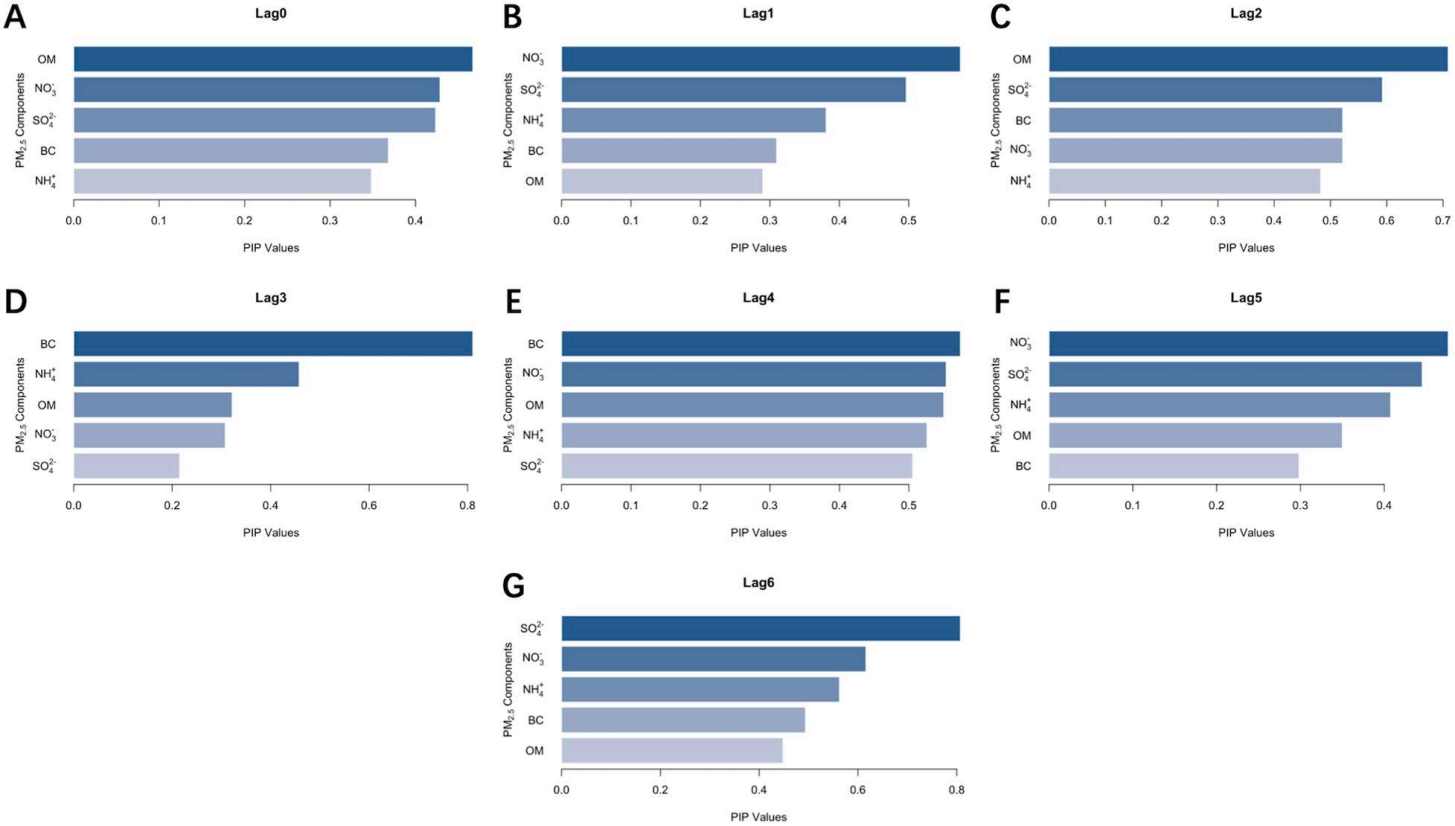
PIP values of BC and other PM₂.₅ components at different lag periods. **(A)** The PIP values of BC and other PM₂.₅ components at a lag of 0 days; **(B)** at a lag of 1 day; **(C)** at a lag of 2 days; **(D)** at a lag of 3 days; **(E)** at a lag of 4 days; **(F)** at a lag of 5 days; **(G)** at a lag of 6 days.

**Figure 8:**
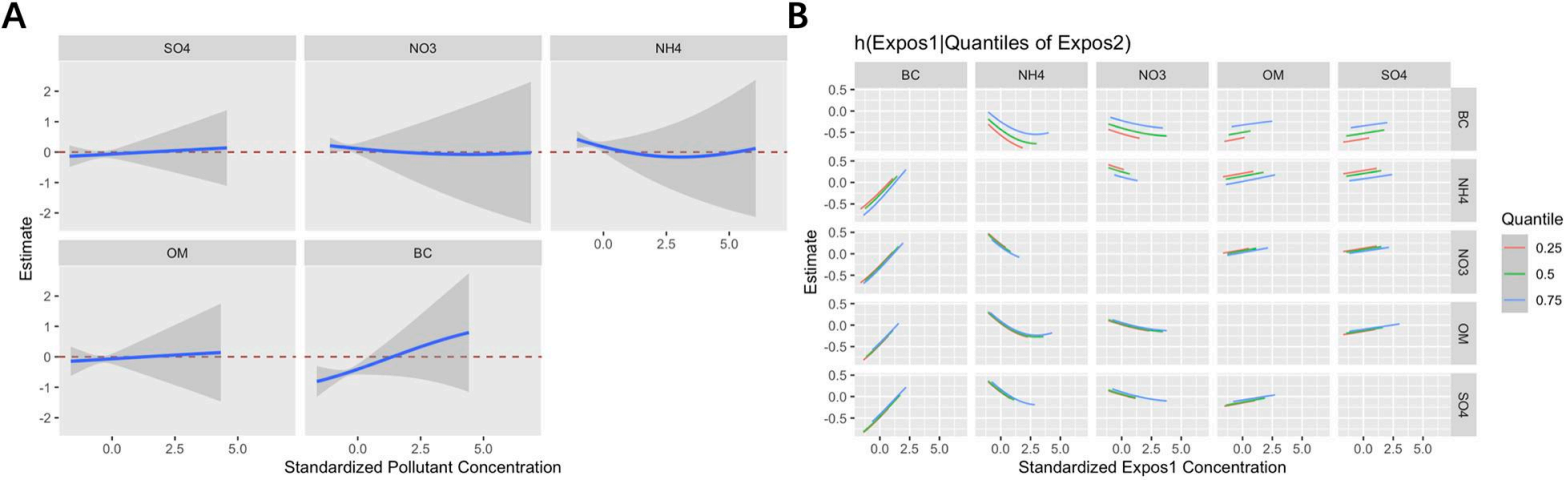
Exposure-response curves and interaction effects of BC and other PM₂.₅ components at a lag of 3 days. **(A)** Exposure-response curves of BC and other PM₂.₅ components with acute asthma exacerbation at a lag of 3 days; **(B)** interaction effects between BC and other PM₂.₅ components at a lag of 3 days.

Further calculation of posterior inclusion probabilities (PIP) indicated that at a 3-day lag, BC (PIP = 0.8106) was the most influential variable associated with acute asthma exacerbations (Figure 7D), followed by NH₄⁺ (PIP = 0.4578), OM (PIP = 0.3216), NO₃⁻ (PIP = 0.3076), and SO₄²⁻ (PIP = 0.2150), with their effects decreasing sequentially. At this lag period, the risk of acute asthma exacerbation increased with rising BC concentrations, and a significant interaction effect was observed between BC and NH₄⁺ (Figure 8).

At other lag periods, NO₃⁻ (PIP = 0.5742) was identified as a key variable influencing acute asthma exacerbations at a 1-day lag (Figure 7B). The risk of exacerbation initially increased and then decreased with rising NO₃⁻ concentrations, and interactions were observed between NO₃⁻ and NH₄⁺/SO₄²⁻ (Supplementary Figure S2). At a 2-day lag, OM (PIP = 0.7090) and SO₄²⁻ (PIP = 0.5922) were the primary influencing factors (Figure 7C). The risk of exacerbation increased with higher OM and SO₄²⁻ concentrations, and interactions were observed between OM and BC/NH₄⁺ (Supplementary Figure S2). At a 6-day lag, SO₄²⁻ (PIP = 0.8070) and NO₃⁻ (PIP = 0.6160) were the most significant contributors (Figure 7G). The risk of exacerbation initially increased and then declined with rising SO₄²⁻ concentrations, while it first decreased and then increased with rising NO₃⁻ concentrations. Additionally, an interaction effect was observed between SO₄²⁻ and NO₃⁻ (Supplementary Figure S2).

Using a multipollutant framework based on BKMR, we evaluated the joint effects of PM₂.₅ components while accounting for their complex correlations. BC showed a positive association with asthma exacerbations within this mixture context. However, given the correlated nature of the pollutants, the estimated effects should be interpreted as part of the overall mixture rather than as strictly independent effects of individual components. The BKMR analysis further suggested potential non-linear and interactive relationships among pollutants. However, these patterns should be interpreted cautiously, as they reflect the joint behavior of correlated exposures within the mixture rather than isolated interaction effects.

### Stratified analysis

**Figure 9:**
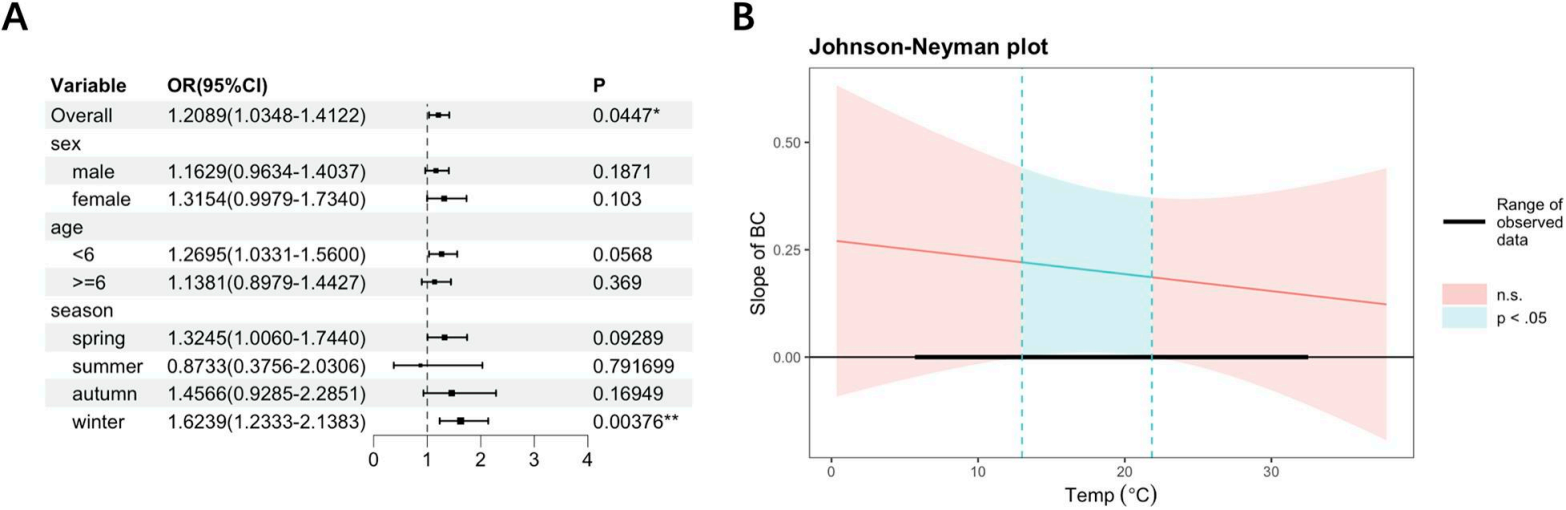
Stratified analysis of the effect of BC on acute asthma exacerbation at lag day 3. **(A)** The stratified analysis of the effect of BC on acute asthma exacerbation at lag day 3; **(B)** the modifying effect of temperature on BC exposure at lag day 3.

Stratified analysis was further conducted on the day with the strongest BC exposure effect (lag day 3), according to age (<6 years old and ≥6 years old), sex, and season of visit (spring, summer, autumn, and winter). No significant associations were observed for age or sex. However, in terms of seasonality, BC exposure in winter was significantly associated with an increased risk of acute asthma exacerbation (aOR = 1.6239, 95% CI: 1.2333–2.1383), whereas no significant associations were found in the other seasons (Figure 9A).

Given the negative correlation between BC and daily average temperature revealed by Spearman analysis, the J-N technique was applied to further investigate potential interaction effects. Results indicated that when the daily average temperature ranged between 13.00°C and 21.85°C, BC exposure was significantly associated with asthma exacerbation, and the risk increased as temperature decreased (Figure 9B).

## Discussion

### BC and acute asthma exacerbation

Our findings indicate that short-term BC exposure is positively correlated with acute exacerbations of childhood asthma, with the greatest effect observed 3 days before exacerbation, consistent with previous studies (10, 27). The COVID-19 pandemic may have influenced healthcare utilization patterns, which could have affected the absolute number of asthma-related visits; however, such effects are unlikely to substantially bias the estimated short-term associations in a case-crossover framework. Importantly, this association remained robust after adjustment for meteorological factors, fever, and PM₂.₅ mass concentration, suggesting that BC exerts an independent effect beyond the overall particulate burden.

In recent years, studies investigating the relationship between BC exposure and acute asthma exacerbations have been increasing. Multiple studies have indicated that BC exposure may aggravate asthma symptoms and elevate the risk of acute exacerbations in children by inducing airway inflammation, oxidative stress, and epigenetic alterations. A study in the United States reported that higher BC exposure within 24 h was positively correlated with fractional exhaled nitric oxide (FeNO) levels in children with asthma, exacerbating airway inflammation without significantly affecting lung function (28). This finding suggests that exposure to BC exacerbates airway inflammation. Given that fractional exhaled nitric oxide (FeNO) is a key biomarker reflecting airway inflammation, its elevation implies that BC may influence the airway microenvironment by promoting the release of inflammatory mediators. However, the study did not observe a significant impact of BC exposure on lung function in children with asthma. This may be attributed to the subtle nature of lung function changes induced by short-term BC exposure or to the influence of individual variability and environmental factors.

Furthermore, BC exposure may influence the onset and progression of asthma through epigenetic regulatory mechanisms. The study by Ji et al. (29) further demonstrated that BC exposure was positively correlated with FeNO levels and negatively correlated with methylation of the nitric oxide synthase 3 (NOS3) gene promoter region. NO plays a critical role in the pathophysiology of asthma, and its excessive expression may exacerbate airway inflammation and hyperresponsiveness, suggesting that BC exposure may aggravate airway inflammation in asthmatic children through epigenetic mechanisms.

Additionally, Oxidative stress may represent one of the key mechanisms through which BC exposure influences asthma. Studies by Mann et al. (30) and Zhang et al. (31) have shown that short-term exposure to traffic-related air pollution during childhood was significantly associated with elevated levels of the oxidative stress biomarker 8-isoprostane, which may exacerbate airway inflammation and hyperresponsiveness through the stimulation of inflammatory cytokines such as interleukin (IL)-6 and tumor necrosis factor (TNF)-α, thereby increasing the risk of acute asthma exacerbation. A study on asthmatic mice revealed that concurrent exposure to BC and a high-humidity environment affected the expression of ATP-binding cassette (ABC) transporters, central carbon metabolism in cancer, resistance to epidermal growth factor receptor (EGFR) tyrosine kinase inhibitors resistance, glioma, and NF-kappa B (NF-κB) signaling pathway in lung tissue (32). Collectively, these studies support a strong association between BC exposure and oxidative stress as well as inflammatory responses.

Previous studies have shown that BC may induce oxidative stress and airway inflammation. These processes typically require 24–72 h to reach a peak inflammatory response, which may explain the delayed effect of BC exposure on asthma exacerbations observed in our study.

### Interaction between BC and PM₂.₅ components

In the two-pollutant models, BC exposure remained significantly associated with asthma exacerbations when co-adjusted with SO₄²⁻, NO₃⁻, and NH₄⁺. These findings suggest that the observed effects of BC are not entirely explained by its correlation with PM₂.₅ and may reflect the contribution of combustion-related particles represented by BC, particularly those originating from traffic emissions. The exclusion of the BC–OM model due to high multicollinearity (VIF > 10) further suggests that BC and OM likely share common emission sources, making it difficult to disentangle their individual effects. Using the Johnson–Neyman approach, we further identified potential interaction effects between BC and secondary inorganic aerosols (SO₄²⁻, NO₃⁻, and NH₄⁺). At lag 3 days, the association between BC exposure and asthma exacerbations was significant within specific concentration ranges of these components, particularly showing an increasing risk with higher SO₄²⁻ levels. This suggests that sulfate-rich environments may modify the health effects of BC, possibly by increasing particle acidity, altering surface chemistry, or facilitating deeper penetration into the respiratory tract. At lag 6 days, significant associations were observed only when SO₄²⁻, NO₃⁻, and NH₄⁺ concentrations exceeded certain thresholds, indicating a potential cumulative or threshold effect under high pollution conditions. These findings highlight that the health effects of BC are not constant but depend on the surrounding chemical composition of particulate matter. Although all VIF values were below 10, moderate collinearity among SO₄²⁻, NO₃⁻, NH₄⁺ and BC may still affect the precision and interpretability of models. Therefore, the findings should be interpreted cautiously.

A key strength of this study is the use of BKMR to account for the complex correlation structure among PM₂.₅ components and to evaluate their joint effects. Unlike traditional regression models, BKMR allows for flexible estimation of potentially non-linear and non-additive relationships within pollutant mixtures. Nevertheless, even within this framework, caution is warranted when interpreting the role of individual pollutants. Due to the inherent correlations among PM₂.₅ components, the estimated exposure–response relationships for specific pollutants, including BC, may reflect their contribution to the overall mixture rather than independent causal effects. Therefore, our findings should be interpreted from a mixture perspective.

Overall, our results suggest that the impact of BC on childhood asthma exacerbations is characterized by temporal lag effects and compositional dependency. The interaction between BC and secondary inorganic aerosols may play a critical role in modulating toxicity, underscoring the importance of considering pollutant mixtures rather than individual components in isolation.

### Other PM₂.₅ components and acute asthma exacerbation

Regarding other PM₂.₅ components (SO₄²⁻, NO₃⁻, NH₄⁺, OM), our single-pollutant model identified a significant association between NH₄⁺ exposure and asthma exacerbation at lag0, though no significant association was observed in the two-pollutant model. In the multi-pollutant model, NO₃⁻ was the primary factor associated with exacerbation at a 1-day lag, with interactions observed between NO₃⁻ and NH₄⁺/SO₄²⁻. At a 2-day lag, OM and SO₄²⁻ were the key contributors, with interactions between OM and BC/NH₄⁺. At a 6-day lag, SO₄²⁻ and NO₃⁻ were the major contributors, with interactions observed between these two components. A Danish birth cohort study (33) demonstrated that prenatal exposure to SO₄²⁻, NO₃⁻, and NH₄⁺, as well as postnatal exposure to SO₄²⁻ and NH₄⁺, were associated with an increased risk of childhood asthma. A Chinese study (34) found that long-term exposure to NO₃⁻ and OM was associated with asthma incidence. Additionally, Ojima et al. (35) reported that exposure to SO₄²⁻ during pregnancy and early childhood was associated with wheezing but not with asthma. To date, most research on PM₂.₅ components has focused on their long-term effects on asthma incidence, while studies on their short-term impact on acute exacerbations remain limited. Further research is needed to elucidate the relationship between exposure to these pollutants and asthma exacerbation, as well as potential interactions and underlying mechanisms.

### Study limitations

Our study has certain limitations and challenges, and its generalizability requires further validation. One important limitation of this study is the use of a unidirectional case-crossover design, in which control periods were selected only from days preceding the event. Although this approach facilitates the assessment of short-term exposure and lagged effects, it may be susceptible to bias arising from long-term temporal trends and seasonal variation in air pollution. In contrast, a time-stratified case-crossover design is generally preferred in air pollution epidemiology, as it better controls for such temporal confounding and avoids overlap bias. Although we adjusted for meteorological factors and co-pollutants in the models, residual confounding related to time trends cannot be completely excluded. Therefore, the findings of this study should be interpreted with caution, and future studies using time-stratified designs are warranted to confirm the robustness of our results. The exclusion of exacerbations attributable to identifiable non-environmental triggers (e.g., allergen exposure, exercise, or medication discontinuation), although intended to reduce confounding, may have introduced selection bias and should be considered when interpreting the findings.

Moreover, compared with studies relying on fixed-site monitoring data, our use of resolution gridded exposure data linked to residential locations may reduce exposure misclassification. However, as TAP data are model-based estimates, some degree of exposure misclassification cannot be excluded. And the spatial resolution of 10 × 10 km may not fully capture local variability in pollutant concentrations, which could result in exposure misclassification and potentially bias the estimated associations toward the null. Future research should incorporate more precise exposure assessment methods, such as personal wearable monitoring devices, to improve the accuracy of BC exposure data.

## Conclusion

In summary, our study, utilizing single-, two-, and multi-pollutant models along with stratified analyses, demonstrated that short-term BC exposure is associated with acute exacerbations of childhood asthma, with the greatest effect observed 3 days before exacerbation. During this lag period, interactions were observed between BC and NH₄⁺. Additionally, BC exposure during winter posed a higher risk for asthma exacerbation. Our study primarily focused on outdoor BC exposure. In real-world conditions, BC exposure does not occur in isolation but rather in combination with other PM₂.₅ components. Moreover, different PM₂.₅ components were found to be associated with asthma exacerbation at different lag periods, suggesting that the mechanisms underlying their effects may differ. Future research should aim to clarify the specific pathways through which BC and other PM₂.₅ components contribute to asthma exacerbation, providing a theoretical basis for clinical prevention strategies.

## Data Availability

The raw data supporting the conclusions of this article will be made available by the authors, without undue reservation.
