# Supplementary material for: Association between short-term exposure to atmospheric black carbon and acute exacerbations of childhood asthma
Source: Front Pediatr. 2026 May 26;14:1756335. doi: 10.3389/fped.2026.1756335 (PMC13246625; doi:10.3389/fped.2026.1756335)
Supplement: Supplementary file 1 [file Table1.docx]

**Supplementary Table 1.** Association between other PM₂.₅ components and acute asthma exacerbation at different lag periods

| **Lag Period** | **Components** | **aOR^a^** | **95% CI** | ***P*** |
| --- | --- | --- | --- | --- |
| 0 | SO₄²⁻ | 1.0199 | 0.9733，1.0687 | 0.489 |
|  | NO₃⁻ | 1.0099 | 0.9641，1.0579 | 0.727 |
|  | NH₄⁺ | 1.0887 | 1.0268，1.1544 | 0.017* |
|  | OM | 0.9572 | 0.9249，0.9907 | 0.036* |
| 1 | SO₄²⁻ | 1.0536 | 1.0057，1.1038 | 0.065 |
|  | NO₃⁻ | 0.9885 | 0.9441，1.0350 | 0.678 |
|  | NH₄⁺ | 1.0185 | 0.9617，1.0786 | 0.599 |
|  | OM | 0.9934 | 0.9608，1.0271 | 0.744 |
| 2 | SO₄²⁻ | 1.0386 | 0.9910，1.0885 | 0.184 |
|  | NO₃⁻ | 1.0338 | 0.9852，1.0848 | 0.257 |
|  | NH₄⁺ | 1.0503 | 0.9895，1.1149 | 0.175 |
|  | OM | 0.9969 | 0.9638，1.0311 | 0.879 |
| 3 | SO₄²⁻ | 1.0046 | 0.9595，1.0518 | 0.871 |
|  | NO₃⁻ | 1.0069 | 0.9616，1.0544 | 0.804 |
|  | NH₄⁺ | 1.0059 | 0.9502，1.0649 | 0.866 |
|  | OM | 1.0276 | 0.9927，1.0637 | 0.194 |
| 4 | SO₄²⁻ | 1.0036 | 0.9579，1.0516 | 0.898 |
|  | NO₃⁻ | 0.9928 | 0.9492，1.0383 | 0.790 |
|  | NH₄⁺ | 1.0004 | 0.9446，1.0594 | 0.991 |
|  | OM | 1.0039 | 0.9704，1.0385 | 0.851 |
| 5 | SO₄²⁻ | 1.0501 | 1.0025，1.1000 | 0.083 |
|  | NO₃⁻ | 1.0152 | 0.9685，1.0641 | 0.598 |
|  | NH₄⁺ | 1.0700 | 1.0089，1.1348 | 0.059 |
|  | OM | 0.9731 | 0.9410，1.0064 | 0.183 |
| 6 | SO₄²⁻ | 1.0076 | 0.9613，1.0561 | 0.791 |
|  | NO₃⁻ | 1.0329 | 0.9842，1.0841 | 0.270 |
|  | NH₄⁺ | 1.0439 | 0.9834，1.1080 | 0.237 |
|  | OM | 0.9841 | 0.9506，1.0189 | 0.449 |
| ^a^aOR, odds ratio adjusted for daily average temperature, relative humidity, fever, and total PM₂.₅ mass.  *, *P* < 0.05. | | | | |
