# Supplementary material for: Association between short-term exposure to atmospheric black carbon and acute exacerbations of childhood asthma
Source: Front Pediatr. 2026 May 26;14:1756335. doi: 10.3389/fped.2026.1756335 (PMC13246625; doi:10.3389/fped.2026.1756335)
Supplement: Supplementary file 2 [file Table2.docx]

**Supplementary Table 2.** Association between two-pollutant exposure and acute asthma exacerbation at different lag periods

| **Lag Period** | **Two-Pollutant**  **(BC+X)** | **BC** | | | **X** | | | | |
| --- | --- | --- | --- | --- | --- | --- | --- | --- | --- |
|  |  | **aOR^a^** | **95% CI** | ***P*** | **aOR** | **95% CI** | | ***P*** | |
| 0 | BC+SO₄²⁻ | 0.8806 | 0.7284，1.0646 | 0.270 | 1.0122 | 0.9570，1.0706 | | 0.306 | |
|  | BC+NO₃⁻ | 0.9649 | 0.8064，1.1544 | 0.743 | 1.0224 | 0.9585，1.0906 | | 0.572 | |
|  | BC+NH₄⁺ | 1.0017 | 0.8373，1.1985 | 0.987 | 1.1043 | 1.0093，1.2083 | | 0.070 | |
|  | BC+OM | 1.2432 | 0.9682，1.5962 | 0.152 | 0.9432 | 0.8895，1.0001 | | 0.101 | |
| 1 | BC+SO₄²⁻ | 0.9409 | 0.7824，1.1316 | 0.587 | 1.0309 | 0.9745，1.0907 | | 0.432 | |
|  | BC+NO₃⁻ | 1.0649 | 0.8987，1.2619 | 0.542 | 1.0107 | 0.9479，1.0777 | | 0.785 | |
|  | BC+NH₄⁺ | 1.0868 | 0.9179，1.2869 | 0.418 | 1.0537 | 0.9653，1.1501 | | 0.326 | |
|  | BC+OM | 1.1707 | 0.9136，1.5001 | 0.296 | 0.9701 | 0.9152，1.0283 | | 0.391 | |
| 2 | BC+SO₄²⁻ | 1.0308 | 0.8523，1.2468 | 0.793 | 1.0288 | 0.9729，1.0880 | | 0.403 | |
|  | BC+NO₃⁻ | 1.1762 | 0.9835，1.4066 | 0.136 | 1.0756 | 1.0079，1.1479 | | 0.065 | |
|  | BC+NH₄⁺ | 1.2137 | 1.0135，1.4535 | 0.077 | 1.1353 | 1.0383，1.2414 | | 0.019* | |
|  | BC+OM | 1.2190 | 0.9414，1.5785 | 0.208 | 0.9586 | 0.9037，1.0168 | | 0.238 | |
| 3 | BC+SO₄²⁻ | 1.1517 | 0.9577，1.3850 | 0.208 | 0.9765 | 0.9242，1.0318 | | 0.477 | |
|  | BC+NO₃⁻ | 1.2421 | 1.0460，1.4750 | 0.038* | 1.0295 | 0.9640，1.0994 | | 0.467 | |
|  | BC+NH₄⁺ | 1.2355 | 1.0416，1.4655 | 0.042* | 1.0320 | 0.9444，1.1277 | | 0.559 | |
|  | BC+OM | 1.2435 | 0.9658，0.9907 | 0.156 | 0.9831 | 0.9266，1.0431 | | 0.341 | |
| 4 | BC+SO₄²⁻ | 0.9725 | 0.8131，1.1632 | 0.798 | 1.0086 | 0.9558，1.0642 | | 0.793 | |
|  | BC+NO₃⁻ | 0.9702 | 0.8218，1.1455 | 0.765 | 0.9987 | 0.9389，1.0623 | | 0.973 | |
|  | BC+NH₄⁺ | 0.9859 | 0.8353，1.1637 | 0.888 | 1.0217 | 0.9386，1.1123 | | 0.677 | |
|  | BC+OM | 0.9052 | 0.7028，1.1660 | 0.518 | 1.0351 | 0.9758，1.0981 | | 0.336 | |
| 5 | BC+SO₄²⁻ | 0.8497 | 0.7125，1.0132 | 0.128 | 1.0299 | 0.9754，1.0874 | | 0.373 | |
|  | BC+NO₃⁻ | 0.9244 | 0.7822，1.0925 | 0.439 | 0.9942 | 0.9309，1.0618 | | 0.884 | |
|  | BC+NH₄⁺ | 0.9589 | 0.8105，1.1343 | 0.681 | 1.0394 | 0.9511，1.1359 | | 0.474 | |
|  | BC+OM | 1.0769 | 0.8406，1.3798 | 0.623 | 0.9616 | 0.9063，1.0202 | | 0.276 | |
| 6 | BC+SO₄²⁻ | 0.8185 | 0.6778，0.9883 | 0.081 | 0.9527 | 0.9008，1.0076 | | 0.155 | |
|  | BC+NO₃⁻ | 0.9152 | 0.7646，1.0955 | 0.418 | 0.9656 | 0.9023，1.0334 | | 0.396 | |
|  | BC+NH₄⁺ | 0.9285 | 0.7755，1.1117 | 0.498 | 0.9475 | 0.8650，1.0378 | | 0.330 | |
|  | BC+OM | 1.0412 | 0.8096，1.3391 | 0.792 | 0.9344 | 0.8790，0.9933 | | 0.068 | |
| ^a^aOR, odds ratio adjusted for daily average temperature, relative humidity, fever, and total PM₂.₅ mass.  *, *P* < 0.05. | | | | | | |  | |  |
