# Supplementary figures and images for: Association between short-term exposure to atmospheric black carbon and acute exacerbations of childhood asthma
Source: Front Pediatr. 2026 May 26;14:1756335. doi: 10.3389/fped.2026.1756335 (PMC13246625; doi:10.3389/fped.2026.1756335)

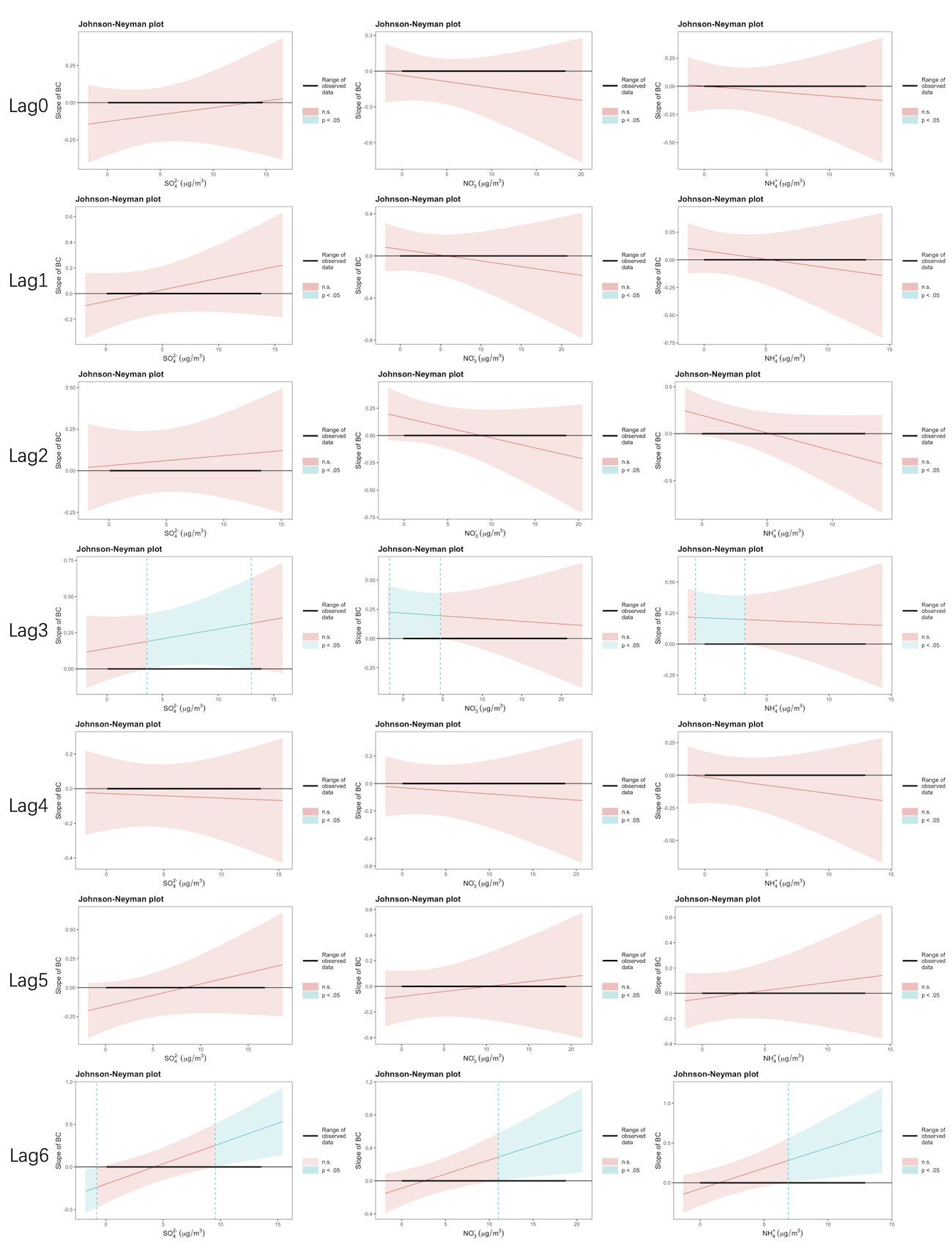

Supplement: Supplementary file 3 [file Image1.tiff]

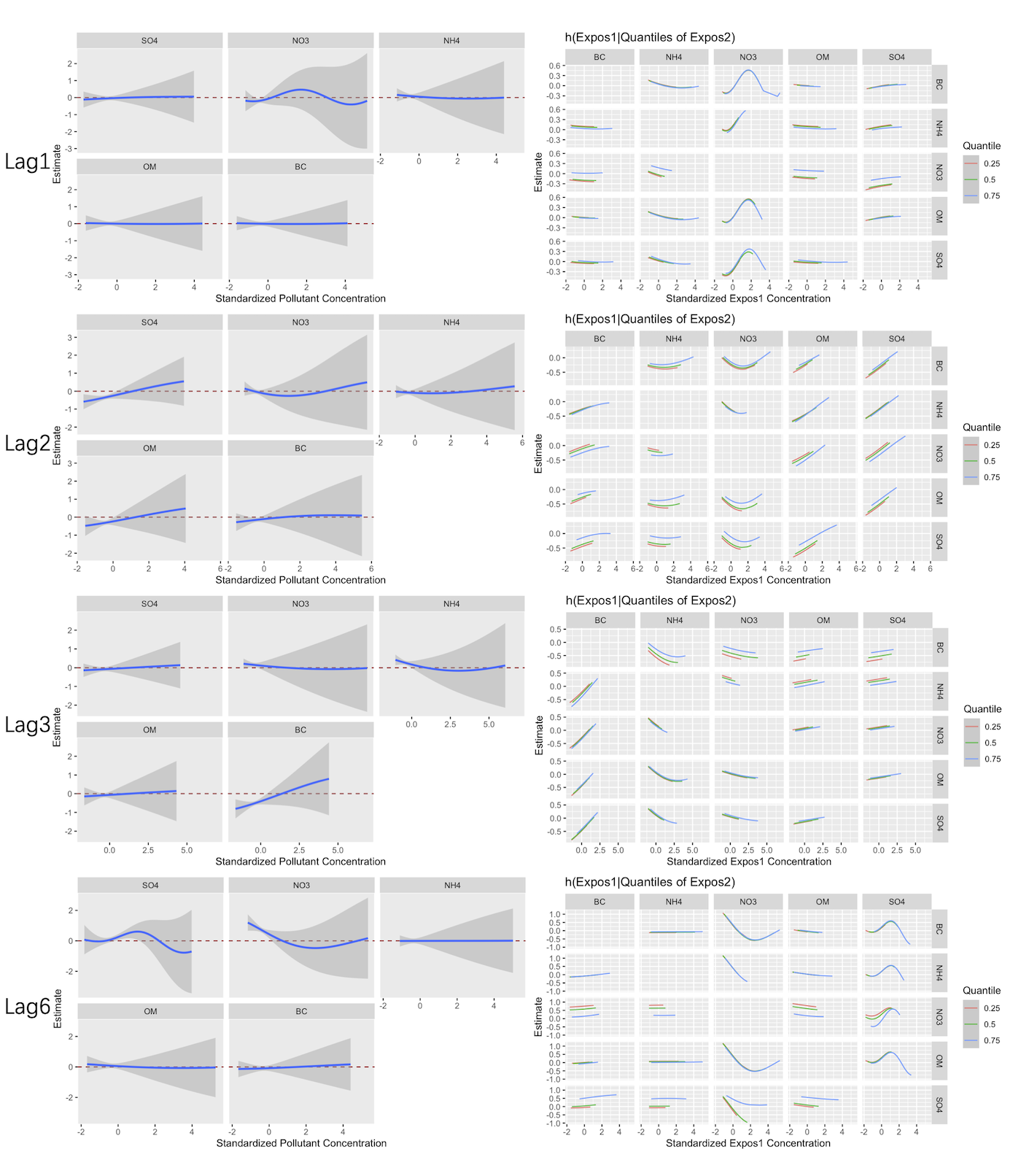

Supplement: Supplementary file 4 [file Image2.tiff]
